# Supplementary material for: Networks of plants: how to measure similarity in vegetable species
Source: Sci Rep. 2016 Jun 7;6:27077. doi: 10.1038/srep27077 (PMC4895227; doi:10.1038/srep27077)
Supplement: Supplementary Information [file srep27077-s1.pdf]

Supplementary Information: Networks of plants: how to measure similarity in vegetable species

Gianna Vivaldo[1], Elisa Masi[2], Camilla Pandolfi[2], Stefano Mancuso[2], and Guido Caldarelli[1,3,4,*]

[1]IMT School for Advanced Studies, Piazza San Francesco 19, 55100 Lucca, Italy
[2]Università di Firenze
[3]London Institute for Mathematical Sciences, 35a South St. Mayfair W1K 2XF London UK
[4]Istituto dei Sistemi Complessi (ISC), Roma, Italy
[*]guido.caldarelli@imtlucca.it

ABSTRACT

Despite the common conception as nearly static organisms. plants do interact continuously with the environment and with each other. It is fair to assume that during their evolution they developed particular features to overcome problems and exploit possibilities. In this paper we introduce various quantitative measures based on recent advancements in complex network theory that allow to measure the effective similarities of various species. By using this approach on the similarity in fruit typology ecological traits we obtain a clear plant classification similar to traditional taxonomic classification. On the other hand by considering diaspore morphological properties we do not find a clear parameter to classify plants species. Complex network theory can then be used in order to determine which feature amongst many can be used to distinguish scope and possibly evolution of plants. Possible uses of this approach range from functional classification to quantitative determination of plant communities in nature.

Families present in the Graph G_1^P

Here we show the structure of families present in the first projection graph where common features are diaspora-based.

Such communities are not homogeneous in terms of family composition (see Fig. 1). Hereafter each cluster composition is summarized, together with the morphological properties that the element families share each other. Notice that one property can be shared by more than a single species in the same cluster, since diaspore morphological features are not mutually exclusive.

- cluster 1 : 884 species (33.21% of database D^3 total species); prevailing families: *Poaceae*, *Fabaceae*, *Rosaceae*, *Plantaginaceae*, *Polygonaceae* (Tab. 1, first column). 709 species have **nutrient** diaspores, followed by 447 showing **flat/wings** diaspore morphology; 204 times is encountered the **elongated** feature.

- cluster 2 : 858 species (32.23%) dominant families: *Asteraceae*, *Cyperaceae*, *Ranunculaceae*, *Rosaceae*, *Apiaceae*, *Apiaceae*, *Amaranthaceae*, *Salicaceae*, *Caprifoliaceae* (Tab. 1, second column). The vast majority of the species (782) show **elongated** diaspore trait; other common observed properties are: **hooked** (220), **ballo/aerenchym** (224), and **flat/wings** (140).

- cluster 3 : 753 species (28.29%), sharing property *no specialization*. Notwithstanding its big dimensions, that cluster is a completely isolated component robust to changes in clustering algorithms. The leading families belonging to cluster cyan are summarized in Tab. 1 (third column). They all share the same **no specialization** property concerning diaspore morphology. That category refers to species whose diaspores can have either a structured surface and no further appendages or specializations (e.g. many *Caryophyllaceae*), or a smooth surface and no further appendages or specializations (e.g. many *Brassicaceae*). Table 1 confirms that behaviour, since *Caryophyllaceae* and *Brassicaceae* are two of the most numerous families with 86 and 43 species each respectively, besides *Orchidaceae* (61) and *Orobanchaceae* (48).

- cluster 4 : 157 species (5.9%); prevailing families: *Brassicaceae*, *Juncaceae*, *Plantaginaceae*, *Asteraceae*, *Lamiaceae*. All these species share **mucilaginous** diaspore property.

- cluster 5 : 9 plants species belonging to *Hydrocharitaceae*, *Brassicaceae*, *Polygonaceae*, and *Araceae* families. They all show **other specialization** concerning diaspore morphology. More in detail, 7 out of 9 are aquatic plants (5 species of

Fam.	cl 1	Fam.	cl 2	Fam.	cl 3
Poaceae	231	Asteraceae	279	Caryophyllaceae	86
Fabaceae	116	Cyperaceae	134	Orchidaceae	61
Rosaceae	66	Ranunculaceae	66	Orobanchaceae	48
Plantaginaceae	30	Rosaceae	54	Brassicaceae	43
Polygonaceae	28	Apiaceae	36	Asteraceae	41
Violaceae	23	Amaranthaceae	34	Apiaceae	38
Apiaceae	22	Salicaceae	32	Rubiaceae	30
Amaranthaceae	20	Caprifoliaceae	27	Primulaceae	29
Juncaceae	17	Potamogetonaceae	23	Campanulaceae	28
Papaveraceae	17	Lamiaceae	21	Saxifragaceae	27
Boraginaceae	16	Onagraceae	21	Lamiaceae	26
Lamiaceae	16	Brassicaceae	20	Crassulaceae	22
Orobanchaceae	16	Boraginaceae	18	Gentianaceae	22
Caryophyllaceae	14	Rubiaceae	9	Rosaceae	22
Ericaceae	14	Typhaceae	8	Plantaginaceae	19
Betulaceae	9	Geraniaceae	7	Amaryllidaceae	18
Caprifoliaceae	9	Plumbaginaceae	7	Ericaceae	18
Pinaceae	9	Alismataceae	6	Scrophulariaceae	14
Santalaceae	9	Caryophyllaceae	6	Convolvulaceae	12
Solanaceae	9	Fabaceae	6	Ranunculaceae	12
Asparagaceae	8	Urticaceae	6	Asparagaceae	11

Table 1. Major families found in $G_{1,P}(N,E)$ clusters 1, 2, and 3 (the largest ones) by modularity (BL) algorithm, and the corresponding number of species belonging to them.

Hydrocharitaceae and 2 of *Araceae* family); 1 species belongs to *Brassicaceae* and 1 to *Polygonaceae*. The 5 species of *Hydrocharitaceae* are strictly related: like other *Hydrocharitaceae*, they are aquatic plants that release their diaspore in water and that, conversely to other plants of the same family, have seeds with very low nutrients content; more, they do not set seeds regularly, preferring asexual reproduction; in both cases (sexual or asexual reproduction) water movements allow the dispersal; the 2 other aquatic (*Araceae*) also prefer asexual reproduction; having no or little roots, the whole plants can float and disperse; the species belonging to the family of *Brassicaceae* has dehishent fruits; finally, the species of *Polygonaceae* rarely produces viable seeds and reproduction is normally asexual (by bulbils)

- **cluster 6**: 1 isolated plant, *X Calammophila baltica Brand (Poaceae)* which doesn't show any of the used morphological properties with the other species.

Table 2 refers to the communities detection results after pruning the graph. Again, detected communities are not homogeneous in terms of family composition. Anyway, more correspondences can be observed between the two panels of Fig 2. Red and cyan clusters, for example, are less heterogeneous, being composed by *Poaceae* and *Rosaceae* families, respectively (white and cerise dots in the right panel). Table 3 reports species and families amount and the corresponding percentage present in each cluster.

It follows a brief description of the four clusters identified by BL method.

- **cluster 1**: 352 species (43.84% of database D^3 total species); *Poaceae* with 228 species are clearly the prevailing family: see white nodes in the right panel of Fig. 2. They are followed by *Juncaceae* (14 plants), *Fabaceae*, *Santalaceae*, *Caprifoliaceae*, *Pinaceae*.
  All these species share that common properties: **nutrients** (315), **flat/wings** (312), **elongated** (240). They do not show (almost most of them) **ballo/aerenchyms** and **mucilaginous** surfaces;

- cluster 2: 345 species (42.96%); dominant families: *Cyperaceae* (89), *Rosaceae* (48), *Ranunculaceae*(42), *Asteraceae*(29). *Cyperaceae* are visible as red dots in Fig. 2 (panel B) in the position corresponding to violet cluster of left panel. That cluster embeds species joined by **elongated** (317) and **hooked** (211) diaspores shape. **Ballo/aerenchyms** and **flat/wings** are shared by 175 and 112 species, respectively. Just 4 species shows **mucilaginous** surfaces;

- cluster 3: 37 species (8.95%); *Rosaceae* family dominates with 23 species, visible as cerise vertices in Fig. 2 (panel B) in the position corresponding to cyan cluster in the left panel. Almost all of them share clearly two properties: **nutrients** and **ballo/aerenchyms** surfaces;

Fam.	cl 1	Fam.	cl 2	Fam.	cl 3	Fam.	cl 4
Poaceae	228	Cyperaceae	89	Rosaceae	23	Potamogetonaceae	20
Juncaceae	14	Rosaceae	48	Cyperaceae	6	Plantaginaceae	19
Fabaceae	11	Ranunculaceae	42	Fabaceae	3	Amaranthaceae	12
Santalaceae	9	Asteraceae	29	Nymphaeaceae	2	Asteraceae	7
Caprifoliaceae	8	Apiaceae	26	Amaranthaceae	1	Brassicaceae	7
Pinaceae	8	Lamiaceae	17	Araceae	1	Juncaceae	3
Polygalaceae	8	Boraginaceae	16	Juncaginaceae	1	Lamiaceae	1
Amaranthaceae	7	Caprifoliaceae	16				
Plumbaginaceae	7	Polygonaceae	10				
Lamiaceae	6	Rubiaceae	9				
Orobanchaceae	6	Geraniaceae	6				
Sapindaceae	6	Alismataceae	5				
Plantaginaceae	4	Typhaceae	4				

Table 2. Families belonging to each of the four clusters identified by communities detection. Graph $G_1^P(N,E)$ is filtered by edges weight $w_{ij} > 1$.

cluster	species	%	families	%
1	352	43.84%	31	27.9%
2	345	42.96%	27	24.32%
3	37	4.61%	7	6.3%
4	69	8.59%	7	6.3%

Table 3. Families and species composition for each cluster detected by BL method on a filtered version of $G_1^P(N,E)$ graph ($w_{ij} > 1$). After filtering just $N = 803$ vertices survive, corresponding each one to a different plant species. The total number of families is equal to 41. Families percentage is referred to the total amount of families into the dataset (111).

- cluster 4 : 69 species (4.61%), dominated by those belonging to *Potamogetonaceae* (20), *Plantaginaceae* (19), and *Amaranthaceae* (12) families. All the species have **mucilaginous** surfaces, some of them show **flat** diaspores (39), in particular species belonging to *Plantaginaceae* and *Juncaceae* families; other individuals show **elongated** diaspore (41), especially *Amaranthaceae*, *Asteraceae*, *Potamogetonaceae*.

Graph of plants $G_2^P(N,E)$ from fruit typology.

As regards the fruit-based graph we have here a short description of the detected communities, together with the main families belonging to them (Tab. 4), and the topological properties of the corresponding species fruits. The graph is shown in Fig. 3.

- cluster 1 : 1426 species belonging to 47 different families, mainly to *Asteraceae* (341), *Poaceae* (231), and *Cyperaceae* (150), *Apiaceae* (95), and *Rosaceae* (84). All these species are characterized by **non fleshy indehiscent fruit** (hard or woody pericarp).
- cluster 2 : 593 species, mainly *Brassicaceae*(116), *Orchidaceae*(61), *Orobanchaceae*(58), *Plantaginaceae*(49), *Fabaceae*(48), all showing **dehiscent fruit with lateral aperture**, i.e. a configuration allowing seeds to be released faster.
- cluster 3 : 326 species, especially *Caryophyllaceae* (99), *Juncaceae*(43), *Primulaceae*(37), *Saxifragaceae*(27), *Crassulaceae*(22). That species are characterized by **dehiscent fruit with upright aperture**, allowing seeds to stay a longer time in the open fruit.
- cluster 4 : 149 species being part of *Rosaceae* (56), *Ericaceae* (11), *Solanaceae* (9), and *Asparagaceae* (7) families, showing **fleshy indescent fruit**.
- cluster 5 : 143 species mainly belonging to *Fabaceae*, *Euphorbiaceae*, *Violaceae*, *Geraniaceae*, and *Brassicaceae* families, all characterized by an **explosive release mechanism**.
- cluster 6 : 13 species subdivided as it follows: 9 belonging to *Pinaceae*, 3 to *Cupressaceae*, and 1 to *Taxaceae* families, respectively. They all share **gymnosperme** seeds with or without hull structures.

Fam.	cl 1	Fam.	cl 2	Fam.	cl 3	Fam.	cl 4
Asteraceae	341	Brassicaceae	116	Caryophyllaceae	99	Rosaceae	56
Poaceae	231	Orchidaceae	61	Juncaceae	43	Ericaceae	11
Cyperaceae	150	Orobanchaceae	58	Primulaceae	37	Solanaceae	9
Apiaceae	95	Plantaginaceae	49	Saxifragaceae	27	Asparagaceae	7
Rosaceae	84	Fabaceae	48	Crassulaceae	22	Caprifoliaceae	7
Lamiaceae	73	Salicaceae	32	Amaryllidaceae	15	Grossulariaceae	7
Ranunculaceae	68	Campanulaceae	30	Amaranthaceae	13	Adoxaceae	6
Amaranthaceae	44	Gentianaceae	27	Ericaceae	13	Araceae	5
Boraginaceae	43	Onagraceae	22	Plantaginaceae	11	Rhamnaceae	5
Rubiaceae	39	Scrophulariaceae	15	Iridaceae	7	Thymelaeaceae	5
Polygonaceae	36	Lentibulariaceae	11	Papaveraceae	6	Hydrocharitaceae	4
Caprifoliaceae	33	Liliaceae	11	Ranunculaceae	6	Nymphaeaceae	4
Potamogetonaceae	24	Asparagaceae	10	Orobanchaceae	5	Cornaceae	3
Fabaceae	23	Hypericaceae	10	Campanulaceae	4	Santalaceae	3
Plantaginaceae	14	Cistaceae	9	Celastraceae	4	Vitaceae	2
Brassicaceae	13	Ranunculaceae	9	Asparagaceae	2	Acoraceae	1
Betulaceae	10	Ericaceae	8	Gentianaceae	2	Amaranthaceae	1
Malvaceae	10	Linaceae	8	Linderniaceae	2	Amaryllidaceae	1
Convolvulaceae	8	Papaveraceae	8	Solanaceae	2	Aquifoliaceae	1
Typhaceae	8	Polygalaceae	8	Tofieldiaceae	2	Araliaceae	1
Fagaceae	7	Droseraceae	5	Butomaceae	1	Berberidaceae	1
Plumbaginaceae	7	Amaryllidaceae	4	Colchicaceae	1	Caryophyllaceae	1
Alismataceae	6	Convolvulaceae	4	Linaceae	1	Dioscoreaceae	1
**Fam.**	**cl 5**	**Fam.**	**cl 6**	**Fam.**	**cl 7**	**Fam.**	**cl 8**
Fabaceae	51	Pinaceae	9	Hydrocharitaceae	5	Cucurbitaceae	2
Euphorbiaceae	24	Cupressaceae	3	Araceae	2		
Violaceae	23	Taxaceae	1	Brassicaceae	1		
Geraniaceae	18			Poaceae	1		
Brassicaceae	14			Polygonaceae	1		
Oxalidaceae	4						
Balsaminaceae	3						
Montiaceae	3						
Apiaceae	1						
Cucurbitaceae	1						
Rutaceae	1						

Table 4. Families belonging to the eight clusters identified by communities detection of graph $G_2^P(N,E)$. That results are robust with respect to changes in detection algorithms.

- cluster 7 : 10 species belonging to *Hydrocharitaceae* (5), *Araceae* (2) and some species belonging to *Brassicaceae*, *Poaceae*, *Polygonaceae* families, mainly. All that species show **not applicable** typology of fruit, typical of those species which either do not produce diaspore or do show vegetative diaspore types.
- cluster 8 : 2 species belonging to *Cucurbitaceae* family: *Bryonia alba L.* and *Bryonia dioica Jacq.*, both showing just **pepo** indehiscent fruit typology.

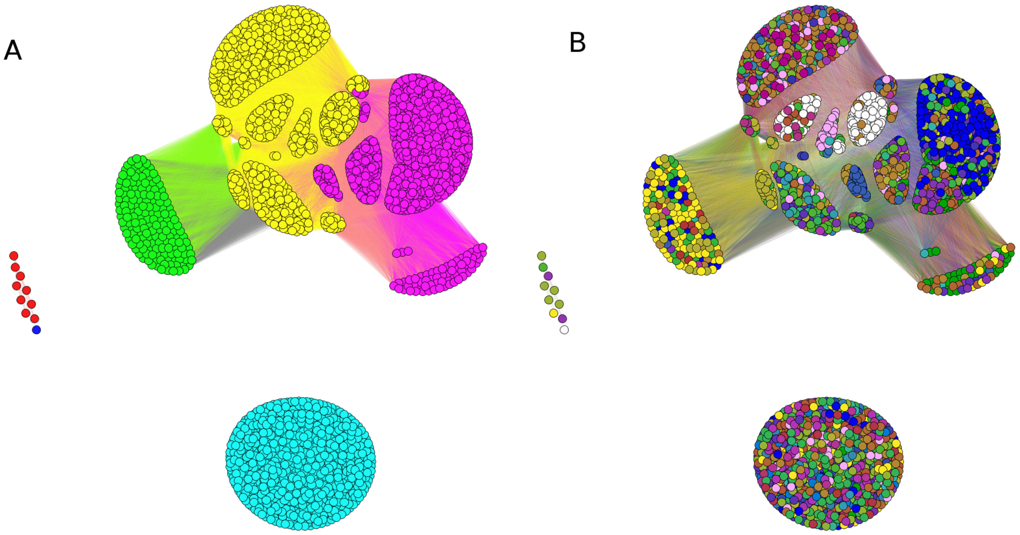

Figure 1. Communities detection based on diaspore morphology. The graphs refers to $G_1^P(N,E)$ communities detection by modularity method. Panel A shows the six communities which are detected: green, yellow, and fuchsia communities are highly connected components. On the contrary, red, blue and cyan clusters are isolated components. While cluster blue just embeds a single species (*X Calammophila baltica Brand*), cluster cyan is quite big, being composed by the 28.29% of total species present in the database D^3, for a total of 12 different families. Panel B shows the families belonging to each cluster. *Asteraceae* (blue, 12.81%), *Poaceae* (white, 8.72%), *Cyperaceae* (dark green, 5.63%), *Brassicaceae* (yellow, 5.41%), *Rosaceae* (cerise, 5.33%) are some of the most numerous. The heterogeneous distribution of families inside each clusters is evident.

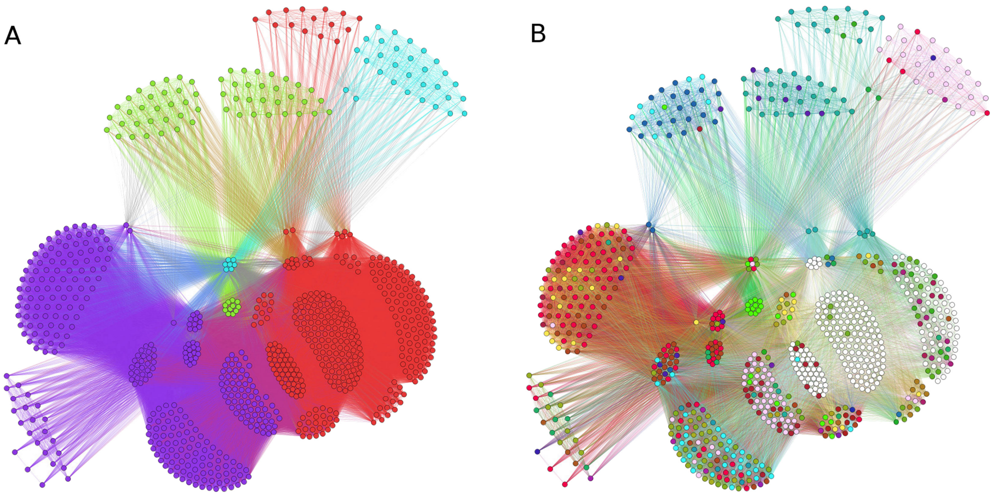

Figure 2. Communities detection on a filtered version of $G_1^P(N,E)$ graph. In that case, edges with weight $w_{ij} = 1$ are removed from the original graph. Four clusters are detected. Clearly each cluster is highly heterogeneous in terms of families composition, but more correspondences are found, and some families begin to dominate some cluster (especially red and cyan clusters of left panel). Prevailing families are visible in panel B: *Poaceae* (white), *Cyperaceae* (red), *Rosaceae* (cerise).

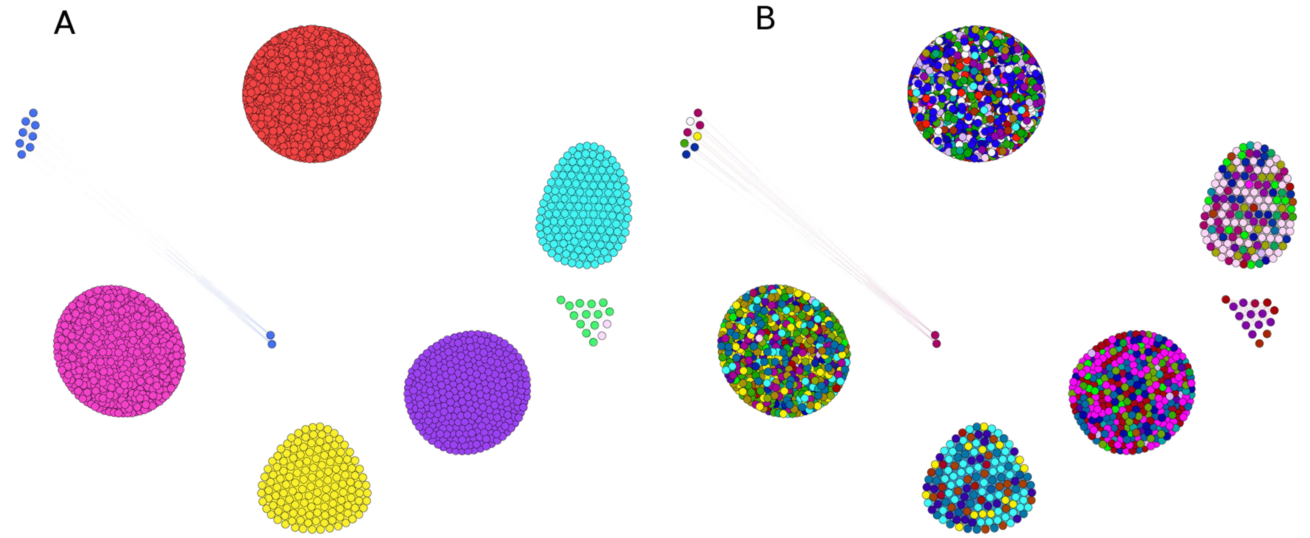

Figure 3. Fruit typology graph communities. $G_P^2(N,E)$ communities detection by modularity method (BL). Only edges with weight $w_{ij} = 1$ are present. Eight isolated communities are detected (panel A), and the corresponding families composition is displayed (panel B). Clearly each cluster is highly heterogeneous in terms of families composition, but not in terms of shared properties between the species belonging to each cluster. A single fruit topological property, in fact, is associated to each cluster and species. Main families are visible: *Poaceae* (white), *Asteraceae* (blue), *Cyperaceae* (red), *Rosaceae* (cerise), *Fabaceae* (cyan), *Caryophyllaceae* (fuchsia).
